# From Paraiyar to Adi-Dravidar: a critical documentary analysis of identity reclamation and persistent caste oppression in Tamil Nadu

**DOI:** 10.3389/fsoc.2026.1785831

**Published:** 2026-08-05

**Authors:** M. Ananthi, S. Prabakar

**Affiliations:** Department of Social Sciences, School of Social Sciences and Languages, Vellore Institute of Technology, Vellore, India

**Keywords:** Adi-Dravidar, caste discrimination, identity reclamation, social exclusion, Tamil Nadu, untouchability

## Abstract

Despite constitutional protections, the 14.4 million Scheduled Caste population in Tamil Nadu, predominantly comprising the Adi-Dravidar community, continues to experience severe systemic oppression. This critical documentary analysis examines the historical trajectory and contemporary lived experiences of the Adi-Dravidar community from 1881 to 2026. Using systematic analysis of 139 secondary sources- including peer-reviewed literature from Scopus, PubMed, and ResearchGate, legal archives, historical documents, and contemporary news reports, this study investigates the gap between symbolic identity reclamation and material social reality. The terminological shift from the pejorative “Paraiyar” to “Adi-Dravidar,” initiated by reformer Iyothee Thass in 1881, marked a significant symbolic victory in asserting indigenous identity. However, findings reveal that 71 documented forms of untouchability practices persist, alongside extreme violence ranging from contamination of water sources to honor killings. Analysis of landmark cases, including the Melavalavu political murders (1997), Vengaivayal water contamination (2022), and multiple honor killings (2003–2025), demonstrates that legislative safeguards remain insufficient. This study argues that symbolic identity transformation, while historically significant, cannot eliminate caste-based oppression without fundamental societal transformation and robust enforcement of anti-discrimination laws. The research contributes to understanding persistent inequality among marginalized populations in India and offers insights for policy interventions addressing structural discrimination.

## 1 Introduction

### 1.1 Background

Caste discrimination in Tamil Nadu has persisted for centuries, extending into the 21st century with lower caste communities continuing to face significant oppression from dominant caste groups (Ranjithkumar, 2024). The historical patterns of caste-based violence and exclusion that have long defined the region’s social fabric remain evident today, reflecting deeply entrenched social hierarchies (Raja et al., 2024). As Ambedkar (1936) articulated, “Caste is not a physical object like a wall of bricks or a line of barbed wire which prevents the Hindus from co-mingling, and which has, therefore, to be pulled down. Caste is a notion; it is a state of the mind.” This conceptualization underscores the complexity of dismantling caste-based oppression, which requires transformation not merely of structures but of consciousness itself.

In India, there are over 1,197 Scheduled Castes and their sub-castes. Tamil Nadu specifically comprises 76 Scheduled Castes, including Pallar, Paraiyar or Adi-Dravidar, and Arundhatiyar (List of Scheduled Caste, 2023). The Scheduled Caste population in Tamil Nadu totals 14,438,445, with 7,204,687 men and 7,233,758 women (Census Digital Library, 2022). The Paraiyars, also known as Adi-Dravidar, constitute the numerical majority among the Scheduled Caste communities in Tamil Nadu (Ravikumar, 2010). The Scheduled Caste community in Tamil Nadu faces at least 71 different forms of untouchability, reflecting the entrenched nature of caste-based discrimination (Sampath, 2023; Alex, 2008). These practices include prohibitions from entering temples (Rajasekaran, 2024), enforcement of the two-tumbler system (Karthikeyan, 2012), and operation of separate ration shops and salons specifically for Scheduled Castes. Discrimination extends to denial of direct water access, restrictions on using bicycles and certain roads (Ramakrishnan, 2021), refusal of postal services to Scheduled Caste households, and compulsory performance during festivals and funerals (Hons, 2018). Children face barriers to school attendance (Kumar, 2021), and government roles often perpetuate marginalization by assigning the Scheduled Castes to sanitary work (Monteiro, 2022).

### 1.2 The Paraiyar community and stigmatization

The Paraiyar community, categorized as part of the Scheduled Caste, has historically been subjected to systemic discrimination by dominant caste Hindu communities, experiencing severe social exclusion and oppression. The term “Pariah,” derived from this community’s name, has become synonymous with “outcast” and “social reject” in English (Definition of PARIAH, 2019). The Merriam-Webster dictionary defines “Pariah” as both an outcast and a member of a low caste in southern India, reflecting its deeply pejorative connotation. This stigmatization is evident in colloquial usage, where individuals who commit errors are derogatorily labelled as “Pariahs” (Mohan, 2018). Consequently, the community’s name has been imbued with a sense of inferiority, reinforcing their marginalized status.

### 1.3 Research gap and significance

While much scholarly research has focused on the broader struggle of the Scheduled Castes for equal rights, the specific efforts of the Paraiyar community to challenge and reclaim their identity have received limited attention. Leaders from within the community have historically contested the derogatory use of the term, arguing that it perpetuates caste-based insult and humiliation (Jayanth, 2022). A comprehensive study of caste struggles necessitates a thorough examination of historical context, as the history of a community provides critical insights into its current social, economic, and political conditions. In the case of the Adi-Dravidar community in Tamil Nadu, understanding their history is indispensable for any research on caste oppression.

This research traces the evolution of the Adi-Dravidar community from its historical roots as an oppressed group to its present-day struggles, shedding light on the enduring impact of caste-based discrimination and the community’s resistance against it. By focusing on their history, this study provides a nuanced understanding of the Adi-Dravidar journey through centuries of social marginalization and their ongoing fight for equality and justice. The specific contribution of this review lies in bringing together three bodies of literature- historical scholarship on identity reclamation, legal and policy analysis of anti-discrimination frameworks, and contemporary documentation of caste atrocities- that are typically examined separately. By synthesizing these strands within a single critical documentary analysis spanning 1881 to 2026, this study is able to show, in a way that single-strand studies cannot, that the persistence of caste-based violence and exclusion runs alongside- rather than despite- symbolic and legislative progress, thereby extending existing caste-studies scholarship on the gap between formal recognition and lived marginalization to the specific case of the Adi-Dravidar community.

### 1.4 Research objectives

This study has three primary objectives:

1. To trace the historical evolution of identity transformation from “Paraiyar” to “Adi-Dravidar” (1881–2026)
2. To document contemporary forms of persistent caste-based discrimination and violence against the Adi-Dravidar community
3. To examine how the lived social realities of the Adi-Dravidar community are represented, interpreted, and documented within existing scholarly literature, legal records, and media reports, in relation to constitutional protections

### 1.5 Paper structure

This paper is organized as follows: Section 2 presents the theoretical framework; Section 3 details the methodology; Section 4 traces the historical evolution of the Paraiyar/Adi-Dravidar identity; Section 5 documents contemporary caste atrocities across multiple dimensions; Section 6 provides analysis and discussion of findings; and Section 7 concludes with policy implications and recommendations.

## 2 Theoretical framework

This section establishes the theoretical foundations guiding the analysis. It first situates the study within Ambedkar’s theorization of caste as a structure reproduced through consciousness rather than physical barriers alone, then draws the distinction between symbolic and material change that organizes the study’s central argument, and finally establishes the intersectional lens through which caste-gender dynamics are analyzed in later sections.

### 2.1 Ambedkar’s theory of caste annihilation

This study is grounded in Dr. B. R. Ambedkar’s theoretical framework on caste as a “state of mind” rather than merely a physical or structural barrier (Ambedkar, 1936). Ambedkar argued that caste operates through internalized hierarchies that reproduce themselves through social practices, religious sanctions, and psychological conditioning. This framework is particularly relevant to understanding why symbolic changes in nomenclature, while important for dignity and self-assertion, may prove insufficient without corresponding transformation in social consciousness and material conditions.

### 2.2 Symbolic vs. material change

The distinction between symbolic and material change forms a central theoretical axis of this study. Symbolic changes- such as renaming from “Paraiyar” to “Adi-Dravidar”- represent important assertions of dignity and historical primacy. However, material change requires transformation in access to resources, political power, social space, and bodily safety. This study examines whether symbolic victories translate into material transformation or remain confined to the realm of nomenclature.

### 2.3 Intersectionality of caste and gender

The study employs an intersectional framework to analyze how caste oppression operates differently across gender lines. The concept of intersectionality originates in Crenshaw’s (1989) analysis of how race and gender combine to produce distinct forms of subordination that neither category explains alone. While Crenshaw’s framework was developed in the context of race and gender in the United States, this study draws more directly on its adaptation to the Indian context by Dalit-feminist scholars, particularly Guru (1995) and Rege (1998), who argue that caste and gender are mutually constitutive rather than additive: Dalit women’s experiences of oppression cannot be understood as caste oppression plus gender oppression, but as a distinct structural position produced by their intersection. Applying this lens, the study examines how caste oppression operates differently across gender lines, particularly evident in honour killings where inter-caste marriages involving Dalit men and upper-caste women face violent repression (Malarvizhi and Thiyagarajan, 2024). This intersection reveals how patriarchal control over women’s sexuality serves to maintain caste boundaries and purity, illustrating Guru’s and Rege’s argument that gender becomes the terrain on which caste hierarchy is policed and reproduced.

## 3 Methodology

This section describes how the study was designed and conducted. It outlines the overall research design and time period (3.1), the sources consulted and the search strategy used to identify them (3.2), the analytical framework applied to the data (3.3), the quality-control measures adopted (3.3.2), the inclusion and exclusion criteria governing source selection (3.4), and the limitations of the approach (3.6).

### 3.1 Research design

This study employs a qualitative research methodology based on critical documentary analysis of secondary data sources. The research examines the historical evolution of the Adi-Dravidar community’s identity and the persistence of caste-based discrimination in Tamil Nadu from 1881 to 2026. Critical documentary analysis allows for systematic examination of how terminology, legal frameworks, and social practices interact across time while revealing the gap between symbolic progress and material reality (see Table 1).

**Table 1:** Research design framework.

Component	Description
Approach	Qualitative, historical-sociological
Method	Critical documentary analysis
Time period	1881–2026
Geographic focus	Tamil Nadu, India
Population	Adi-Dravidar/Paraiyar community (14.4 million)
Data type	Secondary sources (*n* = 139)
Analysis	Thematic, chronological, and case study approaches

### 3.2 Data collection

#### 3.2.1 Data sources

Data were systematically collected from six categories of sources. The collection process involved targeted searches across academic databases, legal archives, historical records, and contemporary media sources.

#### 3.2.2 Search strategy

Systematic searches were conducted using targeted keywords across multiple databases. The search strategy employed both historical terms (Paraiyar, colonial records) and contemporary concepts (honor killings, untouchability practices) to capture the full temporal scope of the study.

#### 3.2.3 Literature selection process

As this study is a critical documentary analysis rather than a formal systematic review, a full PRISMA-style log of every record initially identified and excluded was not maintained during data collection. Table 2 presents an adapted selection-process flow, summarizing the stages through which the final 139 sources were identified, screened, and retained, drawing on the categorical breakdowns already reported in Tables 3, 4.

**Table 2:** Adapted literature selection flow.

Stage	Process
Identification	Records located via keyword searches (Table 4) across Scopus, PubMed, ResearchGate, Google Scholar, legal archives, historical archives, and news archives
Screening	Titles/abstracts or summaries reviewed for relevance to the Adi-Dravidar/Paraiyar community, Tamil Nadu, and caste-based discrimination; duplicates and off-topic records removed
Eligibility	Full sources assessed against the inclusion/exclusion criteria in Table 7 (geographic focus, community specificity, time period, source type, relevance)
Included	One hundred thirty-nine sources retained for analysis, distributed across the source categories in Table 3 and search themes in Table 4

**Table 3:** Data sources and distribution.

source category	specific materials	Number	Purpose
Peer-reviewed journals	Scopus, PubMed, ResearchGate, Google Scholar	67	Theoretical frameworks, empirical studies
Historical documents	Census Reports (1881, 1891, 1961, 2011)	4	Population data, identity classification
Legal documents	SC/ST Act (1989), Constitutional Amendments, Court judgments	15	Legal frameworks, judicial responses
Historical writings	Iyothee Thass’s *Tamilan*, Rettaimalai Srinivasan’s *Paraiyan*	5	Community leaders’ perspectives
News reports	The Hindu, Indian Express, Times of India, Frontline	28	Contemporary atrocity documentation
Scholarly books	Books on caste studies, Dalit movements	12	Contextual analysis
Government reports	NGO reports, official statements	8	Statistical data, official responses
Total		139	

**Table 4:** Search strategy and results.

Theme	Keywords	Databases	Documents
Identity evolution	“Paraiyar,” “Adi-Dravidar,” “Iyothee Thass,” “Rettaimalai Srinivasan”	Google Scholar, ResearchGate, Archives	32
Historical context	“Dravidian movement,” “Tamil Nadu census,” “Colonial records”	Census archives, Academic databases	18
Untouchability	“71 forms untouchability,” “Two-tumbler system,” “Temple entry”	Scopus, News archives	25
Caste violence	“Honor killings,” “Dalit atrocities,” “Inter-caste marriage violence”	Legal databases, News reports	31
Political participation	“Panchayat Raj Dalits,” “Melavalavu murders”	Government reports, Journals	19
Legal framework	“SC/ST Act cases,” “Prevention of Atrocities Act”	Legal archives, Court databases	14
Total			139

### 3.3 Data analysis

#### 3.3.1 Analytical framework

The study employed a five-phase analytical process combining chronological, thematic, and case study approaches. This framework was developed by the author for this study, integrating three established qualitative traditions- historical-chronological mapping, thematic coding, and case study analysis- into a sequential process suited to tracing both the evolution of an identity category over time and its contemporary manifestations Table 5 outlines each phase, the data drawn upon, and its output.

**Table 5:** Five-phase analytical process.

Phase	Procedure	Data used	Output
Phase 1: Historical reconstruction	Chronological mapping	Census data, Reformer writings, colonial records	Timeline (1881–2026) showing identity transformation
phase 2: thematic coding	Categorization into themes	News reports, Court cases, Academic studies	Four major themes identified
Phase 3: Case documentation	In-depth incident analysis	Court judgments, News articles, NGO reports	12 detailed case studies (1997–2025)
Phase 4: Comparative analysis	Contrasting law vs. practice	Legal texts vs. atrocity reports	Gap analysis
Phase 5: Critical synthesis	Pattern interpretation	All sources combined	Main findings and conclusions

#### 3.3.2 Quality control

Multiple quality control measures ensured the reliability and validity of findings (see Table 6).

**Table 6:** Quality assurance measures.

Quality criterion	Implementation	Example
Triangulation	Cross-verification using minimum 3 sources	Melavalavu case verified through Sampath (2023), news reports, Round Table India (2012)
Date accuracy	Verified dates against original reports	Kannagi-Murugesan murder: June 7, 2003 (confirmed PTI, 2022; Ashok and Rupavath, 2022)
Legal authentication	Used official court judgments	SC/ST Act 1989 (Acharya and Acharya, 2020); Constitutional Amendments (Indian Constitution, 2023)
Census validation	Official government websites only	2011 Census: 14,438,445 SC population (Census Digital Library, 2022)
Historical accuracy	Multiple scholarly sources	Iyothee Thass timeline verified (Jeyaraj and Albaris, 2018; Kumar, 2020; Pon Vasanth, 2023)

### 3.4 Inclusion and exclusion criteria

Clear criteria governed source selection to maintain research focus and quality (see Table 7).

**Table 7:** Inclusion and exclusion criteria.

Criteria	Included	Excluded
Geographic	Tamil Nadu specific cases	Other Indian states (unless comparative)
Community	Adi-Dravidar/Paraiyar/SC specific	Other communities (unless interaction)
Time period	1881–2026	Pre-colonial period (before 1881)
Source type	Peer-reviewed, verified news, official documents	Social media, unverified blogs
Relevance	Direct evidence of identity/discrimination	General theory without specificity
Language	English and Tamil	Other languages

### 3.5 Ethical considerations

As this research relied exclusively on publicly available secondary data, it did not involve direct human participants and therefore did not require institutional review board approval. However, ethical rigor was maintained by accurately representing documented incidents without denationalization, acknowledging the lived trauma of affected communities, and properly attributing all sources.

### 3.6 Limitations

This study acknowledges several limitations. First, reliance on documented cases may underrepresent actual incidents due to systematic underreporting, particularly in rural areas. Second, and most importantly, this study relies exclusively on secondary sources; no primary empirical data (interviews, surveys, or fieldwork) were collected from Adi-Dravidar community members. Consequently, the study’s account of “lived social reality” reflects how that reality is represented, interpreted, and documented within existing literature, legal records, and media reports, rather than direct testimony, and findings should be read with this scope in mind. Third, language barriers may have limited access to some Tamil-language sources, though efforts were made to consult Tamil newspapers through English reports and translated academic works. Fourth, historical records may reflect colonial and upper-caste biases, which were addressed through critical reading and cross-referencing with Dalit leaders’ writings.

## 4 Historical evolution: from Paraiyar to Adi-Dravidar

### 4.1 Etymology and early references

The term “Paraiyar” derives from the word “Parai,” referring to a type of frame drum played using a pair of sticks, traditionally passed down through the Paraiyar community (McGilvray, 1983; Roychowdhury, 2024). This drum historically communicated important events such as festivals, meetings, and deaths within villages. However, due to the Paraiyar community’s association with funeral ceremonies, playing the Parai came to be seen as impure within the social construction of caste in Tamil Nadu (Gorringe, 2016). Consequently, the Paraiyar community was relegated to the bottom of the caste hierarchy, a stigma that persisted into the late 1900s and early 2000s (Arun, 2007).

According to French researcher Eleni Varikas (2010), one of the first recorded uses of the word was as “Parea,” referring to a disenfranchised community considered inferior and untouchable. Portuguese navigator Duarte Barbosa, who resided in India from 1,500 to 1,517, documented this term (Mohan, 2018). The English adopted “Pariah” around 1,613, though the Tamil word “Paraya” had much older roots (Roychowdhury, 2024). The earliest known example appears in Sangam-era literature, *Purananuru*, dating to the second or third century CE, suggesting caste discrimination has been practised for approximately 1,800 years. However, Dalit scholar Iyothee Thass challenged the text’s authenticity in 1908, arguing the word could have been added in a later century (Ravikumar, 2005, as cited in Roychowdhury, 2024). The second-oldest mention was found during the Chola Dynasty in the 13th century, with terms “Paraya Cheri” (Paraya settlement) and “Theenda Cheri” (untouchable settlement) indicating these were initially separate categories (Mohan, 2018; Varikas, 2010).

### 4.2 The Dravidian language family and political identity

In 1816, Englishman Francis Whyte Ellis introduced the concept of a Dravidian language family, which Robert Caldwell expanded in 1856, scientifically classifying 12 Dravidian languages beyond the seven initially identified by Zvelebil (1990), Bhadriraju (2024). Major languages, including Tamil, Malayalam, Kannada, and Telugu, were recognized as predating Aryan entry into India (Bhadriraju, 2024). This linguistic classification would later provide the intellectual foundation for political assertions of Dravidian indigeneity.

### 4.3 Iyothee Thass and the birth of “Adi-Dravidar”

The term “Adi-Dravidar” was first introduced by eminent scholar Iyothee Thass from Rayapettai, Chennai, who urged the British government to document Paraiyars as Adi-Dravidar during the first census in 1881 (Kumar, 2020). The term “Adi” signifies “first” or “primordial,” referring to the earliest inhabitants. By addressing the Paraiyar caste people, who had been treated as untouchable, as Adi-Dravidar, Thass emphasized that they were the original people of the land before Aryan entry, initiating an upliftment of their social status.

Iyothee Thass Pandithar, whose original name was Kathuvarayan, adopted his teacher’s name as a mark of respect and recognition (Kumar, 2012). He was a Siddha physician, profound thinker, activist, and polyglot widely recognized for scholarly proficiency in Tamil, English, Pali, and Sanskrit (Ravikumar, 2010). His extensive contributions were dedicated to uplifting the Adi-Dravidar caste, emphasizing social reform and empowerment (Vasanth, 2023).

In 1891, Thass founded the Dravida Mahajana Sabha and organized its first conference in Ooty, where members passed a resolution seeking a law to punish those who called Adi-Dravidar people “pariah” (Kumar, 2020). The Indian National Congress disregarded the organization’s 10 resolutions calling for representation and rights, leading Thass to denounce the Congress as elitist. Nevertheless, Thass became a prominent Dalit activist, using his journal *Oru Paisa Tamilan* (later known as *Tamilan*) as a forum to promote Dalit rights from 1907 until his death in 1914 (Jeyaraj and Albaris, 2018).

### 4.4 Rettaimalai Srinivasan and alternative approaches

Rettaimalai Srinivasan (1859–1945), hailing from Kozhiyalam village in Madras city (Stalin, 2024), played a crucial role in promoting the rights of underrepresented groups. He established the “Paraiyar Mahajana Sabha” in 1891 and organized the second conference addressing the problems of the Paraiyar (Adi-Dravidar) community. He ran the magazine *Paraiyan* from 1893 to 1900, highlighting the community’s struggle (Prabakaran et al., 2023).

Iyothee Thass and Rettaimalai Srinivasan, both from the same community and close relatives, worked toward uplifting their people though they differed in approach. Iyothee Thass advocated for identifying the Paraiyar community as Adi-Dravidar, believing in reclaiming dignity through new identity. In contrast, Rettaimalai Srinivasan initially insisted on retaining the name by which they were insulted, arguing they should confront and rise above the derogatory label (Jayanth, 2022). After Iyothee Thass’s death, Rettaimalai Srinivasan eventually adopted the evolving identities of “Oppressed Classes,” “Adi-Dravidar,” and “Scheduled Caste” as recognized in Indian politics (Leonard, 2017).

### 4.5 M.C. Rajah and legislative recognition

Mailai Chinnathambi Pillai Raja, known as M. C. Rajah (1883–1943), was a Tamil Nadu social activist and politician from the Scheduled Caste who fought for Dalit advancement even before Dr. B. R. Ambedkar (Venkatesh, 2023). In 1922, M. C. Rajah introduced a bill proposing the term “Adi-Dravidar” to replace derogatory labels for communities such as Paraiyans and Pallans, in response to Dalit leaders’ resistance to derogatory names in the late 19th and early 20th centuries (Balasubramaniam, 2016). The bill designated “Adi-Dravidar” for individuals practicing Hinduism and belonging to specific communities, including Paraiyans, Pallans, Valluvans, Malas, and others (Balasubramaniam, 2016).

However, the 1961 census of India categorized these communities differently: Paraiyar, Adi-Dravidar, Samban, Panchama, and Velliyan were grouped as one; Pallan, Devendakulathar, Kuduban, and Kadaiyan formed another; and Arunthathiyar, Chakkiliyan, Madari, and Pagadai were classified as a separate group (Census of India, 1961 Madras, 2015).

### 4.6 The Dravidian political movement

In the mid-20th century, the Dravidian identity took on another political dimension when Periyar E. V. Ramasamy unified the Justice Party and Self-Respect Movement in 1938 to challenge caste oppression. This culminated in the formation of the Dravidar Kazhagam in 1944, marking the rise of the Dravidian political movement aimed at rejecting Brahminical dominance and asserting the rights of Dravidian people in Tamil Nadu (Abhimanyu Arni, 2024).

### 4.7 Legal recognition and contemporary implications

In 1995, a significant incident occurred when Subramanian Swamy labelled Tamil Tigers leader V. Prabhakaran an “international pariah,” igniting political tensions with Chief Minister J. Jayalalithaa. This controversy underscored the term’s degrading implication, reflecting broader societal attitudes toward caste. Both the Madras High Court and Supreme Court have ruled that derogatory caste references against Scheduled Castes constitute an offense under the Scheduled Caste/Scheduled Tribe (Prevention of Atrocities) Act of 1989 (Acharya and Acharya, 2020; Mohan, 2018).

## 5 Contemporary caste atrocities against Adi-Dravidar

### 5.1 Overview of violence

The Scheduled Caste (Dalit) community, including Adi-Dravidar, continues to suffer extreme caste-based atrocities. These include being forced to eat human excrement, having urine passed in their mouths, and enduring sexual abuse (Chakraborty et al., 2023). When conflicts arise with dominant caste groups, retaliation is often severe, involving lynching of Dalit leaders (Kalva, 2021), burning of Dalit villages, and brutal physical punishment. Inter-caste marriages involving Dalits frequently lead to forced suicides or murders to maintain caste purity (Lakshmanan and Srinivasulu, 2017).

The alarming rise in documented crime rates within the Scheduled Caste community in recent years is attributable to a complex interplay of factors, including deep-rooted prejudice, economic inequality, social ostracism, poverty, unemployment, substance abuse, and limited educational opportunities (Downes et al., 2024). Historically, these phenomena were less frequently documented due to limitations in record-keeping, but contemporary heightened awareness has made these issues increasingly apparent (Malhotra, 2025).

### 5.2 Political participation and violence

#### 5.2.1 Constitutional framework

The 73rd and 74th Constitutional Amendment Acts, which came into force in April 1993 and June 1, 1993, respectively, established the Panchayat Raj system in India (Malik, 2024). These amendments mandated reservation of seats for Scheduled Castes and Scheduled Tribes, alongside reserving one-third of positions for women. Following these amendments, Scheduled Castes began participating in the Panchayati Raj system (Sathish, 2019; Indian Constitution, 2023). However, while these legislative measures provided formal framework for inclusion, they also triggered violent backlash from dominant caste groups seeking to maintain their traditional political monopoly.

#### 5.2.2 The Melavalavu murders

Tamil writer Sampath (2023) documented that political participation of Dalits historically was hindered by entrenched caste-based exclusion, only beginning to take shape after the 73rd and 74th Constitutional Amendments (Indian Constitution, 2023). In Melavalavu, Madurai, caste Hindus strongly opposed Dalit participation in politics. After multiple postponements of elections due to resistance, the 1996 local election was finally held under heavy police protection. Murugesan, a Dalit leader, emerged victorious as panchayat president. Despite working for both Dalits and caste Hindus alike, Murugesan and five others were brutally murdered by caste Hindus on June 30, 1997, just 1 year after his election (Sampath, 2023; rti_admin, 2012). This murder reflects intense opposition to Dalit leadership and highlights dangerous realities Dalit leaders face in exercising political power.

The Melavalavu incident is not isolated. Similar prolonged electoral challenges were observed in Keetipatti and Nattaimandalam in Madurai district, where elections were repeatedly disrupted due to caste-based opposition (Sampath, 2023; rti_admin, 2012). These incidents underscore continued marginalization and violence faced by Dalits in attempts to participate in the democratic process.

#### 5.2.3 National political representation

The role of grassroots leadership through the Panchayat system holds significant importance in shaping local governance. However, representation of the Scheduled Caste community, particularly Adi-Dravidar people, in India’s parliamentary bodies, both Rajya Sabha and Lok Sabha, has been historically limited despite their large numbers (Collins, 2017). The Rajya Sabha has 245 seats, and the Lok Sabha has 545 seats, yet Scheduled Caste representation remains minimal (Indian policy, 2023).

During British rule, the Indian political landscape was dominated by upper castes, particularly Brahmins, who were more involved in leadership roles. Even after India’s independence in 1947, political representation gradually expanded only to include members of backward classes, but Scheduled Caste participation, including Adi-Dravidar, remained limited (Sen, 2022). Historical figures like Rettaimalai Srinivasan and Iyothee Thass played crucial roles advocating for Scheduled Caste rights (Ias gyan, 2024; R. Sai Venkatesh, 2024). Together with Dr. B. R. Ambedkar, they submitted proposals and bills aimed at uplifting the Scheduled Caste community (Jayanth, 2022). Despite these efforts, Adi-Dravidar political leaders’ presence in India remains disproportionately low. Ensuring greater representation and leadership roles is essential for achieving more inclusive government (The Politics of Representation, 2012).

### 5.3 Honor killings

#### 5.3.1 Kannagi and Murugesan case (2003)

Kannagi, a 22-year-old from the Vanniyar community, and Murugesan, a 25-year-old from the Adi-Dravidar community, married against their families’ wishes. Their union faced severe opposition, particularly from Kannagi’s family, led by her father C. Duraisamy and brother Maruthupandian. On June 7, 2003, the couple was forcibly taken to their village in Tamil Nadu, where they were brutally murdered in front of villagers. Kannagi’s family members forced poison into their mouths and ears. Both were educated, with Kannagi pursuing B. Com and Murugesan completing engineering. Despite their qualifications, societal pressures and caste prejudices led to their tragic fate. The case was initially mishandled by local police but was later taken over by the Central Bureau of Investigation (PTI, 2022). In 2021, a special court sentenced Maruthupandian to death and others to life imprisonment. However, the Madras High Court later commuted Maruthupandian’s sentence to life and acquitted two co-accused (Ashok and Rupavath, 2022).

#### 5.3.2 Ilavarasan and Divya case (2013)

Ilavarasan, from the Adi-Dravidar community, and Divya, from a caste Hindu community called Vanniyar, married against their families’ wishes, leading to severe consequences. Following their marriage, Divya’s father attempted suicide and died, and caste Hindus retaliated by setting fire to properties in Ilavarasan’s village. On July 11, 2013, Ilavarasan was found dead near a railway track in Dharmapuri, with the CB-CID reporting it as suicide; however, his family did not receive a copy of the report and continues to contest this claim, leaving the true cause of death unclear (Sampath, 2023; Mathew, 2017).

#### 5.3.3 Kausalya and Shankar case (2016)

The inter-caste marriage of Kausalya and Shankar serves as a powerful example of resilience and the fight for justice in the face of caste-based violence. Their public murder, captured on CCTV, highlights the audacity of caste-based honor killings. Kausalya’s father, Chinnasamy, initially sentenced to death, was later acquitted by the Madras High Court in 2020. Five others, also sentenced to death, had their sentences reduced to life imprisonment, while the sessions court acquitted Kausalya’s mother. The weakening judicial response, despite the crime’s brutality, raises concerns about the effectiveness of laws in combating caste violence (Saatvika Radhakrishna, 2024). Despite the unresolved nature of her husband’s murder and deeply entrenched social challenges, Kausalya has transformed her grief into action. By becoming an entrepreneur and activist, she not only fights for her cause but also empowers other women to break free from social constraints. Her establishment of a salon, a profession traditionally reserved for men and lower-caste individuals, symbolizes her defiance against both gender and caste norms (Saatvika Radhakrishna, 2024; Balakrishnan, 2022).

#### 5.3.4 Recent case (2025)

In September 2025, a 28-year-old man from a Scheduled Caste family was brutally murdered by the girl’s brother with a knife, reportedly because the girl belonged to a caste Hindu community. Despite coming from an educated family- his mother is a teacher, and he was employed in an IT company- he was killed (Salahudeen, 2025; Prasitha and Bhuvaneswari, 2025).

#### 5.3.5 Statistical overview of honor killings

A November 2019 report by an NGO working on Dalit and Tribal human rights revealed 195 documented cases of honor killings in Tamil Nadu over the past five years, suggesting a much higher number of unreported cases (Deshpande, 2022). This data underscores deep-rooted societal issues related to caste and social status. The widespread occurrence highlights the urgent need for more focused intervention and legal protections for vulnerable communities.

In this context, marriage and reproduction play crucial roles in perpetuating the caste system. When a woman from a caste Hindu community marries a Dalit, it challenges the traditional caste hierarchy, as offspring from such unions do not fit neatly within the rigid caste framework. This suggests that caste is not just a social construct but also biologically reinforced through endogamy. The continuation of caste relies heavily on preserving caste purity through reproductive practices. Thus, inter-caste marriages, particularly involving Dalits, have the potential to disrupt the caste structure (Deshpande, 2022).

### 5.4 Untouchability practices

#### 5.4.1 The Thinniyam incident (2002)

In May 2002, in Thinniyam village, Tiruchirapalli District, Rajalakshmi Subramanian, an upper-caste leader from the caste Hindu (Kallar) community, solicited ₹2,000 from the Adi-Dravidar community with the promise of providing housing. After three years of unfulfilled promises, on May 20, 2002, at 10:30 a.m., the Adi-Dravidar community protested by beating drums, demanding their money back. In response, caste Hindus violently attacked two men from the Adi-Dravidar community, Ramasamy and Murugesan. The two were brutally beaten and subjected to a grotesque act of humiliation, forced to feed human excrement to one another. This horrifying act symbolized deeply entrenched caste-based dominance, using extreme measures to reinforce social hierarchies. Although some members of the caste Hindus were arrested, the Adi-Dravidar community faced economic backlash, with many losing their jobs in the village (SV, 2002; Padmini Sivarajah, 2019). This case reflects ongoing caste opposition faced by marginalized communities in rural Tamil Nadu, illustrating how caste Hindus maintain dominance over marginalized groups.

#### 5.4.2 The Vengaivayal atrocity (2022)

In December 2022, the village of Vengaivayal in Pudukkottai district witnessed severe caste Hindu discrimination against the Adi-Dravidar community. Human excrement was deliberately mixed into their water tank, leading to widespread illness. District Collector Kavitha Ramu investigated and found that, in addition to water contamination, a two-tumbler system was being enforced at a local tea shop, where caste Hindu individuals used silver tumblers and Adi-Dravidar people were given copper ones. The Adi-Dravidar community was also banned from entering the village temple (Rajendran, 2022). Although arrests were made concerning the two-tumbler system, the issue of water contamination was not adequately resolved (ANI, 2023). In a significant act of defiance against caste Hindu exclusion, Collector Kavitha personally took members of the Adi-Dravidar community into the temple, challenging discriminatory practices (Sneha Belcin, 2023). These actions exemplify the extreme and degrading measures employed to enforce caste boundaries and perpetuate oppression (Sampath, 2023).

Despite the existence of the Untouchability Offences Act of 1995 (Pratim Sarkar, 2024), which was intended to combat caste-based discrimination, Adi-Dravidar communities continue to face significant challenges. Numerous incidents of caste discrimination still occur in Tamil Nadu. This persistence is due to some individuals’ lack of awareness about the law and its associated punishments, while others simply disregard it. Additionally, there are those with limited access to justice, further exacerbating the issue.

#### 5.4.3 Forms of untouchability

Table 8 categorizes the 71 documented forms of untouchability practices that persist in Tamil Nadu, organized by domain of discrimination.

**Table 8:** Forms of untouchability practiced against Adi-Dravidar in Tamil Nadu.

Category	Specific practices	Evidence sources
Spatial segregation	Separate settlements (Paraya Cheri), restricted roads, denied housing	Mohan (2018), Varikas (2010), Ramakrishnan (2021)
Religious exclusion	Temple entry prohibition, separate worship spaces	Rajasekaran (2024), Belcin (2023)
Economic discrimination	Forced sanitary work, job denial, separate ration shops	Monteiro (2022), Sampath (2023)
Social humiliation	Two-tumbler system, separate salons, forced drum playing at funerals	Hons (2018), Karthikeyan (2012), Sampath (2023)
Access denial	Water source contamination, denied direct water access, bicycle prohibition	Ramakrishnan (2021), Rajendran (2022)
Educational barriers	Children barred from schools, separate seating arrangements	Kumar (2021)
Service discrimination	Post office delivery denial, separate service counters	Sampath (2023)
Violence	Lynching, honor killings, physical assault, sexual abuse, forced consumption of excrement	Chakraborty et al. (2023), Kalva (2021), Lakshmanan and Srinivasulu (2017), Sampath (2023)

### 5.5 Legal framework and implementation gap

Widespread discrimination against Dalits led to the 1989 Prevention of Atrocities Act (Acharya and Acharya, 2020), which criminalized severe abuses like public humiliation, land seizure, and destruction of property. This law aimed to protect Dalit rights and prevent caste-based violence (Manas, 2014). Despite the presence of numerous laws and punitive measures designed to curb caste-based discrimination, the persistence of such practices indicates that legislative frameworks alone are insufficient to eradicate social inequality. The root cause lies in the absence of humanity and mutual respect among individuals, especially in their treatment of others outside their immediate family circles. While laws can instil fear of punishment, in many regions they are either inadequately enforced or circumvented by those in power. Moreover, there are cases where local governments, instead of protecting marginalized communities, actively perpetuate untouchability through discriminatory practices.

## 6 Analysis and discussion

### 6.1 The paradox of symbolic change

This study reveals a fundamental paradox at the heart of the Adi-Dravidar experience: while the community achieved significant symbolic victory through identity reclamation, this transformation has not translated into material improvement in social status or reduction in violence. The shift in terminology from “Paraiyar” to “Adi-Dravidar,” initiated by Iyothee Thass in 1881 and institutionalized through M. C. Rajah’s legislative efforts in 1922, represented an important assertion of dignity and historical primacy. By claiming status as “Adi” (first/original) Dravidians, the community challenged the hierarchical positioning that relegated them to the bottom of the caste system. In the terms established in Section 2.2, this was a symbolic victory of the highest order- yet, as the findings below demonstrate, it remained confined to the realm of nomenclature and dignity, without the corresponding material transformation in resources, political power, social space, and bodily safety that the symbolic/material distinction anticipates.

However, the persistence of 71 forms of untouchability, documented honor killings spanning two decades (2003–2025), and political violence such as the Melavalavu murders (1997) demonstrate that symbolic recognition does not automatically dismantle structural oppression. This finding supports Ambedkar (1936) theoretical insight that caste operates as a “state of mind” rather than merely a structural arrangement. Changing nomenclature addresses the structural level of categorization but leaves untouched the psychological and social mechanisms that reproduce caste hierarchies through daily interactions, spatial segregation, and violence.

### 6.2 Persistence of violence despite constitutional protections

The documented cases reveal a troubling pattern: constitutional amendments designed to enable political participation (73rd and 74th Amendments, 1993) and laws designed to prevent atrocities (SC/ST Act, 1989) have proven insufficient to protect Adi-Dravidar individuals from extreme violence. The Melavalavu case exemplifies this gap, despite constitutional reservation ensuring Murugesan’s election as panchayat president, he and five others were murdered within 1 year of assuming office (Sampath, 2023). This suggests that formal political rights, when granted to communities experiencing deep social subordination, may actually increase vulnerability to violent backlash.

Similarly, the honor killings documented in this study––from Kannagi-Murugesan (2003) to the recent Mayiladuthurai case (2025) reveal that legal prohibitions against caste-based violence have failed to prevent murders of Dalit men who marry women from dominant castes. The judicial outcomes in these cases further indicate systemic failure: in the Kausalya-Shankar case, despite CCTV evidence of murder, the primary accused (Kausalya’s father) was acquitted on appeal, and death sentences were reduced to life imprisonment (Saatvika Radhakrishna, 2024). This pattern suggests that the legal system itself may be influenced by caste hierarchies, resulting in inadequate protection and justice for victims.

### 6.3 Intersectionality of caste and gender

The honor killing cases analyzed in this study reveal how caste oppression operates through control of women’s sexuality and reproductive choices. When women from dominant castes marry Dalit men, they challenge two systems simultaneously: the caste system’s prohibition on inter-caste marriage and the patriarchal system’s control over women’s marital choices. The violent response, from forced feeding of poison (Kannagi-Murugesan) to public murder (Kausalya-Shankar), demonstrates that maintaining caste boundaries is deeply intertwined with patriarchal control.

Notably, the cases involving Dalit men marrying dominant caste women face more severe and public violence compared to the reverse situation. This asymmetry suggests that upper-caste male control over “their” women’s reproductive capacity is central to maintaining caste purity. As offspring inherit their father’s caste, a dominant caste woman marrying a Dalit man produces Dalit children, representing a perceived loss to the dominant caste. This analysis aligns with the Dalit-feminist intersectional framework (Guru, 1995; Rege, 1998; Malarvizhi and Thiyagarajan, 2024) that demonstrates how caste and gender operate as mutually constitutive, rather than additive, systems of oppression.

### 6.4 The water contamination incident: symbolic and material violence

The Vengaivayal water contamination incident (2022) merits particular attention as it demonstrates how caste-based violence targets the most basic human needs. Deliberately mixing human excrement into the Adi-Dravidar community’s water supply represents both symbolic degradation (associating the community with pollution) and material harm (causing widespread illness). The incident also revealed multiple forms of discrimination operating simultaneously: water contamination, the two-tumbler system in tea shops, and temple entry prohibition (Rajendran, 2022; Belcin, 2023).

The lack of accountability, authorities failed to identify culprits for water contamination despite arresting individuals for the two-tumbler system, reflects a wider problem: a justice system that often fails to protect the most marginalized. This selective enforcement suggests that certain forms of discrimination (public two-tumbler system) are more visible and therefore subject to intervention, while others (water contamination in segregated settlements) remain invisible to authorities or are implicitly tolerated.

### 6.5 Political representation and the limits of electoral democracy

This study’s findings on political participation reveal a critical limitation of electoral democracy in contexts of deep social hierarchy. The 73rd and 74th Constitutional Amendments (1993) mandated political reservation for Scheduled Castes in local governance, creating formal political equality (Indian Constitution, 2023). However, the Melavalavu case demonstrates that dominant castes responded to this formal equality with violence, essentially nullifying constitutional guarantees through murder.

At the national level, despite comprising 14.4 million people in Tamil Nadu alone (Census Digital Library, 2022), Adi-Dravidar representation in the Rajya Sabha and Lok Sabha remains minimal (Collins, 2017). This underrepresentation is partially explained by historical factors, during British rule and early post-independence period, political participation was dominated by upper castes, particularly Brahmins (Sen, 2022). However, the persistence of this pattern suggests ongoing structural barriers to political mobilization and representation.

The role of early political leaders like Iyothee Thass, Rettaimalai Srinivasan, and M. C. Rajah in advocating for Scheduled Caste rights (Jayanth, 2022; Ias gyan, 2024; Venkatesh, 2024) demonstrates that political consciousness and organization within the community existed from the late 19th century onward. The limited translation of this consciousness into substantial political power suggests that electoral mechanisms alone cannot overcome entrenched social hierarchies.

### 6.6 Comparison with broader Dalit studies

This study’s findings on the Adi-Dravidar community align with broader patterns documented in Dalit studies literature but also reveal specific dynamics. The persistence of untouchability practices despite legal prohibition mirrors findings across India (Manas, 2014). However, the Tamil Nadu context reveals particular intensities: 71 documented forms of untouchability (Sampath, 2023) represent systematic and comprehensive social exclusion. The honor killings data, 195 documented cases over 5 years, according to one NGO report (Deshpande, 2022), suggests Tamil Nadu may experience particularly high rates of violence related to inter-caste marriage.

The Dravidian political movement’s role in this context merits consideration. While Periyar E. V. Ramasamy and the Dravidar Kazhagam challenged Brahminical dominance and caste oppression (Arni, 2024), the persistence of severe discrimination against Adi-Dravidar suggests that anti-Brahmin politics did not necessarily translate into anti-caste politics more broadly. Dominant non-Brahmin castes (such as Vanniyar and Kallar) appear as primary perpetrators in the cases documented in this study, suggesting that the dismantling of Brahminical privilege did not eliminate caste hierarchies but potentially reconfigured them.

### 6.7 Implications for social change

This study’s findings suggest that meaningful social change for the Adi-Dravidar community requires transformation at multiple levels simultaneously:

#### 6.7.1 Psychological level

As Ambedkar (1936) argued, caste operates as a “state of mind.” Changing this requires not merely legal prohibition but fundamental transformation in how individuals conceptualize human dignity and equality. The continued practice of untouchability despite legal prohibition indicates that many individuals have not internalized principles of equality.

#### 6.7.2 Social level

Daily practices of discrimination- from the two-tumbler system to spatial segregation- must be actively dismantled through both enforcement and social mobilization. The role of District Collector Kavitha Ramu in personally escorting Adi-Dravidar individuals into the temple in Vengaivayal (Belcin, 2023) demonstrates how official action can challenge discriminatory practices, though such interventions remain exceptional rather than systematic.

#### 6.7.3 Institutional Level

The justice system’s inadequate response to caste violence- from weak prosecutions to sentence reductions on appeal- indicates need for systemic reform. Fast-track courts, mandatory training on caste discrimination for judges and police, and accountability mechanisms for inadequate enforcement are essential.

#### 6.7.4 Economic Level

Economic dependency reinforces caste hierarchies. When Adi-Dravidar individuals challenged discrimination in Thinniyam, they faced economic retaliation through job loss (SV, 2002). Economic independence and diversification of livelihood options are therefore crucial for enabling resistance to discrimination.

#### 6.7.5 Political Level

Representation in governance must extend beyond numerical presence to substantive power. The murder of elected Dalit leaders demonstrates that formal political rights require protection through security measures and swift prosecution of political violence.

## 7 Conclusion

### 7.1 Summary of key findings

This critical documentary analysis of the Adi-Dravidar community in Tamil Nadu from 1881 to 2026 reveals a profound paradox: significant symbolic achievement in identity reclamation has not translated into material improvement in social status or reduction in violence. The study identified four key findings:

First, the terminological shift from “Paraiyar” to “Adi-Dravidar,” initiated by Iyothee Thass in 1881 and institutionalized through M. C. Rajah’s legislative efforts in 1922, represented an important symbolic victory in asserting dignity and historical primacy as original inhabitants. This achievement demonstrated community agency in resisting stigmatization and reclaiming identity.

Second, despite this symbolic transformation and despite constitutional protections (73rd and 74th Amendments) and anti-discrimination laws (SC/ST Act 1989), 71 documented forms of untouchability persist in Tamil Nadu, ranging from spatial segregation to denial of basic services. Contemporary cases documented in this study- including water contamination in Vengaivayal (2022), the two-tumbler system in Madurai villages, and temple entry prohibitions- demonstrate that discriminatory practices remain deeply embedded in social structures.

Third, extreme violence against Adi-Dravidar individuals continues, manifested in political murders (Melavalavu, 1997), honor killings (spanning 2003–2025), and grotesque acts of humiliation (forced consumption of human excrement in Thinniyam, 2002). The persistence of such violence, documented through 195 honor killings over 5 years according to NGO reports (Deshpande, 2022), indicates that legal prohibitions have proven insufficient to prevent caste-based atrocities.

Fourth, analysis of judicial outcomes reveals systematic failure in delivering justice. The acquittal or sentence reduction in multiple honor killing cases (Kannagi-Murugesan, Kausalya-Shankar) despite clear evidence, combined with lack of accountability in untouchability cases (Vengaivayal water contamination), suggests that the legal system itself may be influenced by caste hierarchies.

### 7.2 Theoretical contributions

This study contributes to theoretical understanding of caste oppression by demonstrating the insufficiency of symbolic change in the absence of material transformation. While nomenclature matters for dignity and self-conception, changing how a community is named does not automatically change how that community is treated. This finding supports Ambedkar (1936) conceptualization of caste as a “state of mind” that requires psychological transformation, not merely legal or terminological change.

The study also contributes to intersectional analysis by demonstrating how caste oppression operates through control of women’s sexuality and reproductive choices. Honor killings represent enforcement of caste boundaries through patriarchal violence, revealing how systems of oppression are mutually constitutive rather than operating independently.

### 7.3 Policy implications

Based on this study’s findings, several policy interventions are recommended:

#### 7.3.1 Enforcement mechanisms

Strengthen enforcement of existing anti-discrimination laws through: (1) mandatory fast-track courts for caste atrocity cases with strict timelines; (2) accountability measures for police and officials who fail to register complaints or investigate cases; (3) protection schemes for inter-caste couples including safe houses and legal assistance; (4) security provisions for elected Dalit political leaders.

#### 7.3.2 Educational interventions

Implement mandatory caste sensitization in school curricula from primary level onward, focusing on Indian Constitution’s principles of equality, historical injustices faced by Scheduled Castes, and contemporary manifestations of discrimination. Teacher training on recognizing and addressing caste-based discrimination in classrooms is essential.

#### 7.3.3 Monitoring and documentation

Establish systematic monitoring of the 71 forms of untouchability documented in Tamil Nadu through: (1) regular surveys in villages and towns; (2) accessible reporting mechanisms for victims; (3) civil society engagement in documentation and advocacy; (4) public disclosure of discrimination data to create accountability.

#### 7.3.4 Economic empowerment

Expand economic opportunities for Scheduled Castes through: (1) entrepreneurship support programs; (2) skill development aligned with market demands; (3) reservation in private sector employment; (4) land redistribution and agricultural support. Economic independence reduces vulnerability to discrimination and enables resistance.

#### 7.3.5 Social transformation

While legislative and policy measures are necessary, they are insufficient without broader social transformation. This requires: (1) civil society mobilization challenging discriminatory attitudes; (2) inter-caste dialogue initiatives; (3) celebration of Dalit cultural contributions; (4) media representation challenging stereotypes; (5) support for inter-caste marriages through social acceptance programs.

### 7.4 Limitations and future research

This study’s reliance on secondary data limits access to direct community voices and lived experiences. Future research should employ ethnographic methods to capture everyday experiences of discrimination and resistance. Additionally, the focus on documented cases likely underrepresents actual incidents due to systematic underreporting, particularly in rural areas. Survey research could provide more comprehensive data on prevalence of discriminatory practices. In particular, community-based participatory research- involving Adi-Dravidar residents and organizations as co-researchers rather than only as subjects of study- could validate, complicate, or extend the patterns identified here through this literature synthesis, while also centering the community’s own framing of its history and priorities.

Future research directions include: (1) research examining how other communities- including dominant-caste groups and other Scheduled Caste communities in Tamil Nadu- perceive and justify honor killings and caste-based violence against Adi-Dravidar people, to understand the social attitudes that sustain such violence; (2) longitudinal studies tracking changes over time in specific villages or regions; (3) investigation of successful resistance movements and conditions enabling community mobilization; (4) analysis of role of education in challenging or reproducing caste hierarchies; (5) examination of caste dynamics in urban versus rural contexts.

### 7.5 Final reflections

This study demonstrates that India’s progress toward social equality remains incomplete. The Adi-Dravidar community’s experience reveals that constitutional guarantees, legislative protections, and symbolic recognition, while necessary, are insufficient to eliminate centuries of oppression. The persistence of extreme violence, from honor killings to forced consumption of excrement, and systematic discrimination in access to water, temples, and public spaces indicates that caste hierarchies remain deeply embedded in social consciousness and daily practices.

The historical trajectory traced in this study- from Iyothee Thass’s assertion of Adi-Dravidar identity in 1881 to contemporary atrocities in 2026- spans 145 years. This temporal expanse reveals both continuity and change: continuity in the forms of oppression (untouchability, violence, exclusion) and change in the community’s self-conception and political organization. The question facing Indian society is whether another century will pass with the same patterns of violence and discrimination, or whether genuine transformation can be achieved.

Achieving genuine equality requires more than legal reform or symbolic recognition. It demands, as Ambedkar argued, transformation of the “state of mind” that sustains caste hierarchies. This transformation requires concerted action at multiple levels: individual psychological change through education and interaction, social mobilization challenging discriminatory practices, institutional reforms ensuring accountability, economic redistribution reducing dependency, and political representation enabling voice and agency.

The struggle of the Adi-Dravidar community is not merely their own but reflects broader questions about India’s commitment to the constitutional values of liberty, equality, and fraternity. Honoring these principles requires not just acknowledging historical injustices but actively dismantling the structures and mindsets that perpetuate them. Only through such comprehensive transformation can the symbolic victory of identity reclamation translate into material reality of dignity, safety, and equality for all.
